# Cutaneous verrucous carcinoma: A clinicopathological study of 21 cases with long-term clinical follow-up

**DOI:** 10.3389/fonc.2022.953932

**Published:** 2022-10-13

**Authors:** Qian Ye, Li Hu, Meng Jia, Li-Jia Deng, Sheng Fang

**Affiliations:** Department of Dermatology, The First Affiliated Hospital of Chongqing Medical University, Chongqing, China

**Keywords:** cutaneous, verrucous carcinoma, clinicopathologic, differential, squamous cell carcinoma

## Abstract

**Background:**

Cutaneous verrucous carcinoma (CVC) is a rare variant of squamous cell carcinoma and sometimes shares similar clinical and histopathological features with other verrucous lesions.

**Methods:**

We performed a retrospective study of 21 patients diagnosed with CVC between 2012 and 2022 by reviewing clinical and histopathological data. We also compared the clinicopathological features of patients with CVC, giant condyloma acuminatum (GCA), and pseudoepitheliomatous hyperplasia (PEH). We obtained follow-up data by reviewing medical records and telephone interviews.

**Results:**

The average age of patients with CVC was 63.6 years, with a male predominance. The location of disease is mostly found in the foot, followed by the lower legs. Histologically, CVC is characterized by an exo-endophytic growth pattern with severe keratinization and a blunted rete ridge with pushing margins. Clinical features including exudation and crusting, induration, irregular borders, and warty surface, as well as pathological features including growth pattern, koilocytotic cells, depth and morphologic features of acanthosis, severe keratinization, and degree of dermal inflammation, were significant in distinguishing cutaneous CVC, GCA, and PEH.

**Conclusion:**

Identification of the clinicopathological features is essential to distinguish CVC from its mimics and to make an early diagnosis. Because of the potential for recurrence and metastasis, CVC requires aggressive treatment.

## Introduction

Cutaneous verrucous carcinoma (CVC) is a rare variant of well-differentiated squamous cell carcinoma (SCC) characterized by slow growth and low metastatic potential (1). However, it can be locally aggressive, and recurrence is not uncommon. Tumors typically develop in older adults (usually patients 60–70 years of age) and predominantly in Caucasian men (2). These lesions are sometimes indistinguishable from giant condyloma acuminatum (GCA) and pseudoepitheliomatous hyperplasia (PEH) (3), which often delay diagnosis and make treatment more difficult. Although immunohistochemical studies of verrucous carcinoma have been reported in a number of publications, there is still a lack of validated and reliable immunohistochemical markers (1, 4–6). To date, there are no critical molecular biomarkers that are sensitive enough to discriminate reliably between them. The diagnosis of CVC remains difficult and relies largely on clinical features and histopathological characteristics (7). Over the last 20 years, only sporadic cases of CVC have been reported, and a series of studies are still lacking (8–11). To shed light on this issue, we investigated the clinicopathological features of CVC and compared them with those of GCA and PEH for differential diagnosis to better characterize CVC and its mimics and facilitate diagnosis and treatment. Moreover, the follow-up data were obtained by review of the medical records or telephone contact to describe the clinical outcomes.

## Materials and methods

### Materials

A retrospective review of patients seen at the Department of Dermatology in the First Affiliated Hospital of Chongqing Medical University, Chongqing, China, between January 2012 and January 2022 was conducted. All slides stained with hematoxylin and eosin (H&E) were taken from the Dermatological Department of the First Affiliated Hospital of Chongqing Medical University. All patients with a histologically confirmed diagnosis of CVC were included in this study. Additionally, the same number of patients with GCA and PEH was randomly selected. The clinical data were collected from inpatient or outpatient medical records and pathology application forms. All cases had complete and detailed medical records. The prognosis data were obtained using follow-up information by a review of the medical records or telephone contact. Ethics approval was granted by the Ethics Committee of the First Affiliated Hospital of Chongqing Medical University (2022-150). Informed written consent was obtained from the study participants prior to the study.

### Methods

All patients were identified through a computer-generated search using the term “cutaneous verrucous carcinoma” in the pathology results from the database of the Department of Dermatology in the First Affiliated Hospital of Chongqing Medical University (n = 21). All pathologic records of patients with the keywords “giant condyloma acuminatum” and “pseudoepitheliomatous hyperplasia” were also retrieved and reviewed retrospectively in a standard computerized database. We retrospectively analyzed the sex, age, location, prognosis, and clinical and pathological characteristics of CVC and compared them with GCA and PEH. A clinical and pathological review of the lesions was performed independently by two dermatologists and one pathologist who specialized in dermatopathology and were blinded to the original diagnoses when they examined the histopathological features to minimize bias. Individuals were screened *via* a telephone interview conducted by a dermatologist or a study nurse.

### Statistical analysis

Descriptive analyses were performed to evaluate clinical data, including sex, age, duration, anatomic distribution, dimension, and clinical aspects of CVC, as well as pathological data. Moreover, we also compared CVC, GCA, and PEH in terms of clinicopathological characteristics. Comparisons of categorical variables were conducted using the *x* ^2^ test and Fisher’s exact test. A p-value less than 0.05 was considered statistically significant. Statistical analyses were performed using the statistical software SPSS for Windows version 23.0.

## Results

### Clinical data

**Figure 1:**
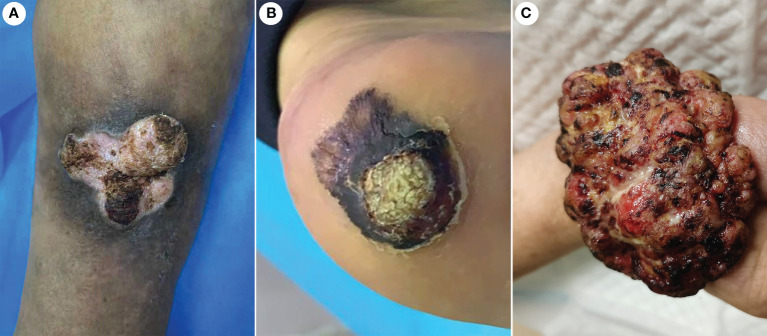
Gross features of CVC. **(A)** A warty plaque with an irregular border on the right lower leg. **(B)** A verrucous mass in the left foot. **(C)** A large verrucous mass in the right hand. CVC, cutaneous verrucous carcinoma.

The incidence of CVC was approximately 0.09% in outpatients who have undergone biopsies. A total of 21 CVC cases were collected, including 17 men and four women. Epidemiologic and clinical characteristics are summarized in **Table 1**. Patient ages ranged from 45 to 84 years (mean 63.6 years). The clinical presentation was a mass in 13 cases, and those presenting as plaques were in eight cases (**Figure 1**). Tumor size ranged from 1.6 to 6.2 cm, with more than half of the patients having lesions (11 cases) larger than 3.5 cm in diameter. The most common site of onset was the foot, seen in 10 patients, followed by the lower legs in six patients. It occurred in the hand in two patients and in the buttocks, prepuce, and thigh in one case each. More than half of the patients (13/21 cases) had a disease duration of more than 1 year. All lesions had exudation and crusting on the surface and showed an induration with irregular borders, with 19 patients having a warty surface. In addition, more than half of the patients had ulcerations on the lesions. There were 15 cases with accompanying itching and/or pain.

**Table 1:** Epidemiologic and clinical aspects of verrucous carcinoma.

Clinical characteristics n	Verrucous carcinoma (n = 21)
Sex	M = 17
	F = 4
	M/F ratio = 4.3
Age	63.6 ± 12.1 (45–84)
Duration	
<6 months	4
6–12 months	3
>12 months	13
Unknown	1
Anatomic distribution	
Foot	10
Lower leg	6
Hand	2
Prepuce	1
Buttock	1
Thigh	1
Largest dimension	
<2 cm	3
2–3.5 cm	**7**
>3.5 cm	11
Clinical aspects	
Exudation and crusting	21
Induration	21
Irregular border	21
Warty surface	19
Ulceration	12
Itching and/or pain	15
Gross findings	
Mass	13
Plaque	8

### Pathology data

**Figure 2:**
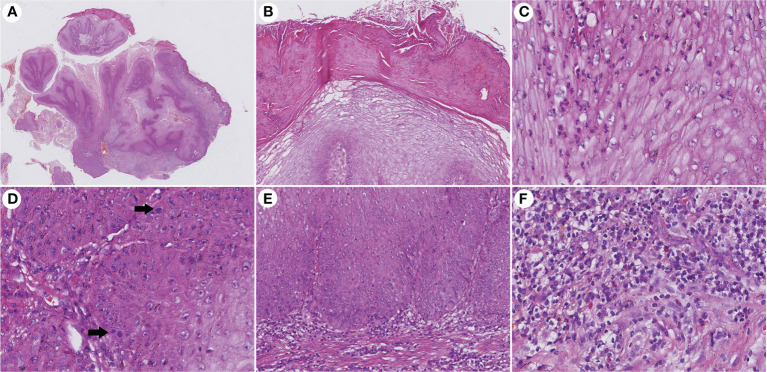
Histopathological changes in CVC. **(A)** Low magnification showed an exo-endophytic growth pattern (H&E, ×10). **(B)** Exophytic component showed prominent parakeratosis and exudation without koilocytotic cells (H&E, ×100). **(C)** Intraepithelial microabscesses in severe keratinization tumor cells (H&E, ×400). **(D)** Basal cell atypia and mitosis (arrow) can be observed (H&E, ×400). **(E)** Blunted rete ridge with pushing margins (H&E, ×200). **(F)** Severe dermal inflammation of lymphocytes, plasma cells, and eosinophils (H&E, ×400). CVC, cutaneous verrucous carcinoma.

The histopathological features of CVC often presented as a verrucous or hyperkeratotic surface with a deep endophytic (21/21 cases) and broad blunted rete ridge (21/21 cases), which pushed down the basement membrane rather than infiltrate into the dermis (21/21 cases) (**Figure 2**). Exophytic components with papillomatosis often showed a diminished granular layer (16/21) with prominent parakeratosis (18/21 cases) and exudation (21/21). Large spiny keratinocytes contain abundant eosinophilic cytoplasm and enlarged nuclei. Dyskeratotic cells (21/21 cases) were observed, while koilocytotic cells were not present. The tumor cells are well differentiated with severe keratinization (21/21 cases), while basal cell mild atypia (10/21 cases) is often confined to one or two layers of the tumor base with rare mitoses. Mild basal cell atypia is defined as increased nuclear size, nuclear size variability, nuclear irregularities, and nuclear hyperchromasia. Intraepithelial microabscesses (17/21 cases) associated with keratin pearls (9/21 cases) can be seen in the CVC, which may serve as a clue to the diagnosis. Blunt acanthosis constitutes asymmetry of the silhouette (21/21 cases) of CVC, with reactive hyperplasia (16/21 cases) seen on the sides of the non-invasive epithelium adjacent to the tumor as irregular acanthosis with elongated rete. The dermal papillae immediately adjacent to the pushing edge are often filled with varying degrees of inflammatory cells with an edematous stroma and dilated capillaries. Fibrotic collagen (13/21 cases) in the dermis is common. The histopathological findings are summarized in **Table 2**.

**Table 2:** Summary of pathologic data.

Characteristic	Total sample
Exo-endophytic growth pattern	21/21 (100)
Intraepithelial microabscesses	17/21 (81)
Marked parakeratosis	18/21 (86)
Diminished granular layer	16/21 (76)
Dyskeratotic cells	21/21 (100)
Spongiosis	21/21 (100)
Keratin pearls	9/21 (43)
Asymmetrical silhouette	21/21 (100)
Blunted rete ridge with pushing margins	21/21 (100)
Basal cell mild atypia	10/21 (48)
Micro infiltration	7/21 (33)
Well-differentiated with severe keratinization	21/21 (100)
Dermal fibrosis	13/21 (62)
Reactive hyperplasia of adjacent epidermis	16/21 (76)
Degree of dermal inflammation	
Mild	0/21 (0)
Moderate	5/21 (24)
Severe	16/21 (76)
Types of inflammatory cells	
Prominent neutrophils	11/21 (52)
Prominent lymphocytes	21/21 (100)
Prominent eosinophils	18/21 (86)

### Comparison of the clinicopathological features of cutaneous verrucous carcinoma and its mimics

**Figure 3:**
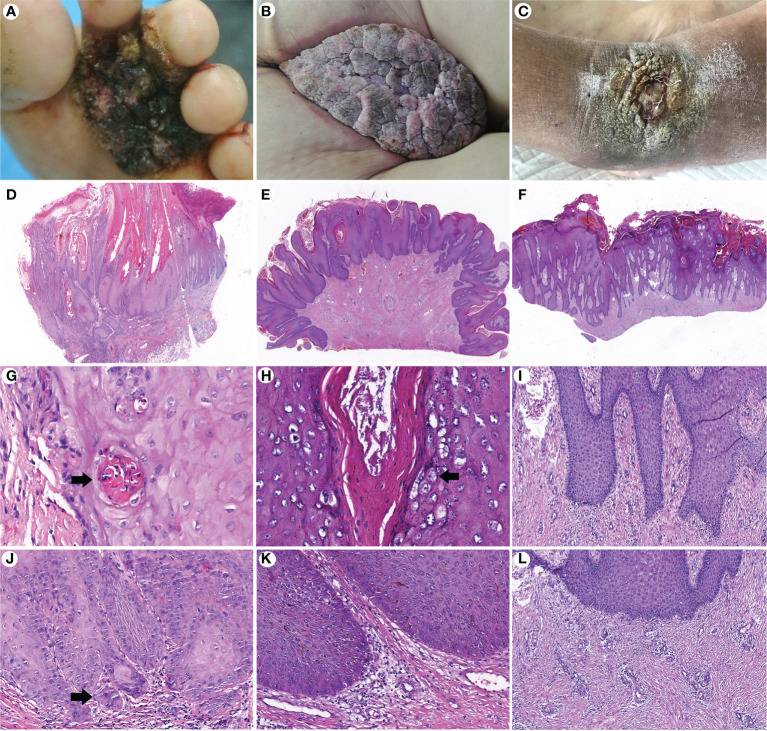
Comparison of clinicopathological features of cutaneous verrucous carcinoma **(A, D, G, J)**, giant condyloma acuminatum **(B, E, H, K)**, and pseudoepitheliomatous hyperplasia **(C, F, I, L)**. **(A)** A verrucous mass in the forefoot. **(B)** A large cauliflower-like mass in the perianal area. **(C)** A verrucous plaque in the right dorsal foot. **(D)** An exo-endophytic growth pattern with pushing margins (H&E, ×10). **(E)** An exophytic growth pattern (H&E, ×10). **(F)** An endophytic growth pattern (H&E, ×10). **(G)** Well-differentiated keratinocytes with severe keratinization and intraepithelial microabscesses (arrow) (H&E, ×400). **(H)** Koilocytotic cells in the granular layer (arrow) (H&E, ×400). **(I)** More basophilic and irregular acanthosis (H&E, ×200). **(J)** Basal cell atypia with microinvasion (arrow) (H&E, ×200). **(K)** Mild dermal inflammation infiltration without basal cell atypia (H&E, ×200). **(L)** Perivascular lymphocytic infiltration (H&E, ×200).

GCA and PEH are benign lesions that can mimic verrucous carcinoma clinically and microscopically. A total of 42 (21 GCA and 21 PEH) cases were collected for comparing histopathological features with CVC. The clinicopathological data are summarized in **Table 3**. Exudation and crusting, induration, irregular borders, and warty surface were significantly more prevalent in CVC than in GCA and PEH. The pathological manifestations, such as growth pattern, koilocytotic cells, depth of acanthosis, morphologic features of acanthosis, severe keratinization, and degree of dermal inflammation, played a significant role in differentiating CVC, GCA, and PEH (**Figure 3**). The exo-endophytic growth pattern, invasion of the reticular dermis, blunt acanthosis, and severe dermal inflammation were more prevalent in CVC, whereas the exophytic growth pattern and papillomatous acanthosis were significantly related to GCA. In addition, endophytic growth patterns and irregular acanthosis were more frequent in PEH. Notably, koilocytotic cells (p < 0.01) were significantly more common in GCA rather than in CVC and PEH, which is an important clue for the diagnosis of GCA. Basal cell mild atypia was more associated with CVC and PEH rather than GCA.

**Table 3:** Clinicopathological features of cutaneous CVC, GCA, and PEH.

Clinicopathological features	VC, N = 21, n (%)	GCA, N = 21, n (%)	PEH, N = 21, n (%)	Value of p
Exudation and crusting	21/21 (100)	10/21 (48)	14/21 (67)	p < 0.01
Induration	21/21 (100)	3/21 (14)	16/21 (76)	p < 0.01
Irregular borders	21/21 (100)	7/21 (33)	17/21 (81)	p < 0.01
Warty surface	19/21 (90)	21/21 (100)	5/21 (24)	p < 0.01
Growth pattern				p < 0.01
Endophytic	0/21 (0)	0/21 (0)	18/21 (86)	
Exophytic	0/21 (0)	21/21 (100)	0/21 (0)	
Exo-endophytic	21/21 (100)	0/21 (0)	3/21 (14)	
Koilocytotic cells	0/21 (0)	19/21 (90)	1/21 (5)	p < 0.01
The depth of acanthosis				p < 0.01
Papillary dermis	0/21 (0)	19/21 (90)	18/21 (86)	
Reticular dermis	21/21 (100)	2/21 (10)	3/21 (14)	
Morphologic features of acanthosis				p < 0.01
Blunt with pushing margins	18/21 (86)	1/21 (5)	2/21 (10)	
Papillomatous	0/21 (0)	17/21 (81)	1/21 (5)	
Irregular	3/21 (14)	3/21 (14)	18/21 (86)	
Severe keratinization	21/21 (100)	2/21 (10)	3/21 (14)	p < 0.01
Degree of dermal inflammation				p < 0.01
Mild	0/21 (0)	11/21 (52)	3/21 (14)	
Moderate	5/21 (24)	7/21 (33)	11/21 (52)	
Severe	16/21 (76)	3/21 (14)	7/21 (33)	
Basal cell mild atypia	10/21 (48)	2/21 (10)	9/21 (43)	p = 0.017

CVC, cutaneous verrucous carcinoma; GCA, giant condyloma acuminatum; PEH, pseudoepitheliomatous hyperplasia.

### Follow-up

All patients underwent surgery, and postoperative biopsy showed negative margins. The median postoperative follow-up time was 42 months (range, 4–65 months). Two patients presented with local recurrence (located in the hand and foot). Both recurrences occurred within 1 year after surgery. No patients had regional lymph nodes or distant metastases.

## Discussion

Verrucous carcinoma (VC) is a rare, low-grade, and well-differentiated SCC with the potential for metastasis but with a predilection for localized destruction (10). VC usually involves the mouth, throat, esophagus, and skin (7). It is classified into four types based on the location of occurrence, including 1) oral–gastrointestinal VC (oral florid papilloma and Ackerman’s tumor), 2) anal–genital VC, 3) foot VC, and 4) verrucous carcinoma of other skin sites (12). VC commonly occurs in the mucosa, such as the oropharynx and genital mucosa (12, 13). CVC can appear anywhere on the skin, most commonly on the foot (14). CVC has only been reported as uncommon or rare, with no accurate incidence (15, 16). To study the clinicopathological characteristics of CVC, we retrospectively analyzed a cohort of CVC patients from a tertiary care center over the last 10 years.

The clinical presentation of CVC is often a slow-growing warty plaque or mass (7), which is often misdiagnosed or delayed until a skin biopsy is performed to confirm the diagnosis. More than half of the patients in our study had a disease course of more than 1 year. In our series, the results were consistent with previous studies showing that CVC occurs mostly in elderly individuals, with a male predominance (16). In contrast to classical SCC (17), CVC is rarely seen at the site of exposure, and none of the patients in this study had lesions on the head or face. This suggests that there might be differences in the pathogenesis of CVC and SCC and that ultraviolet B (UV-B) is not as important in the pathogenesis of CVC as it is in SCC (18). The pathogenesis of verrucous carcinoma remains unclear, but several contributing factors have been proposed, including trauma, chronic inflammation, and poor local hygiene (19, 20). Penera et al. reported that verrucous carcinoma usually occurs in weight-bearing areas, and thus, the possibility of pressure damage as an etiology is increased (21). Although previous literature has reported a low frequency of lower leg involvement in CVC (22), in our series, there were six of 21 cases that occurred in the lower leg, the second most common site of the disease. The occurrence of this area is often thought to be associated with chronic venous ulcers (22). We noted that most patients had dark red basal and crusted exudative surfaces on the lower leg lesions, suggesting that chronic inflammation plays an important role in the development of CVC. Although the number of cases in this study was small, we would like to draw attention to the warty masses and plaques on the lower leg in clinical practice, especially in patients with chronic venous ulcers. The skin of the lower legs is also susceptible to infection, which is often caused by poor local hygiene and minor trauma (21). We observed that almost all lesions in the case series presented as induration and irregular borders, which could be explained by the nature of localized aggressiveness (2). In three cases, single or multiple nodules were observed around primary CVC lesions, resembling satellite lesions, which were less evident in GCA and PEH.

The histological criteria for the diagnosis of CVC include the exo-endophytic growth pattern with a deep and broad blunted rete ridge with pushing margins (16, 23). These criteria are compatible with the outcomes of the study. We also observed that asymmetrical silhouette, basal cell mild atypia, well-differentiated tumor cells with keratinization, dermal fibrosis, reactive hyperplasia of adjacent epidermis, and eosinophilic infiltration were common in CVC. According to previous literature, dermal fibrosis may result from the stimulation of persistent inflammation, which may accelerate tumor invasion and growth due to the limited regenerative capacity of the skin dermis (24, 25). We noticed common eosinophilic infiltration in the dermis in addition to previously documented lymphocytic infiltration (16, 23). Eosinophils are able to regulate tumor progression by interacting with tumor cells or shaping the tumor microenvironment (26). They can secrete soluble mediators that promote angiogenesis and matrix remodeling, thereby promoting tumor growth (27). The relationship between eosinophils and the local destruction of CVC still requires further study.

CVC should be clinically differentiated from other diseases in a similar pattern (3). We compared the clinicopathological features of patients diagnosed with CVC with those of patients with GCA and PEH. We observed that CVC and PEH presented clinically as exudation and crusting, induration, and irregular borders more commonly than GCA, while warty surfaces were more common in CVC and GCA than in PEH. The exo-endophytic growth pattern, blunt acanthosis with pushing margins, invasion of the reticular dermis, and severe dermal inflammation were significantly more prevalent in CVC (2). In addition to the exophytic growth pattern, papillomatous acanthosis restricted to the papillary dermis was significantly associated with GCA (28). The literature has shown that PEH is associated with prolonged inflammation and/or chronic infection, as well as many cutaneous neoplasms (29), but whether PEH is a precursor to CVC remains controversial (30). Koilocytotic cells were significantly more common in GCA than in VA and PEH, which is an important clue for differential diagnosis (31).

The locally aggressive and metastatic nature of CVC has led to surgical resection as the recommended treatment. Photodynamic therapy and CO_2_ laser have been reported to be used preoperatively to reduce tumor size. Other treatment modalities include chemotherapy, immunotherapy, cryosurgery, and intradermal injection of interferon-α. The contribution of radiotherapy has been controversial due to the risk of anaplastic transformation (11). In our series, two patients who were followed up experienced local recurrence. Both recurrences occurred within 1 year after surgery, which is largely consistent with the findings of Koch et al. (11) Studies have shown that SCC tumors with a diameter greater than 2 cm have a threefold increase in their metastatic rate (23). When the tumor invasion depth is greater than 6 mm, the metastasis rate is as high as 16% (32, 33). However, the sites of our two recurrent patients were located in the foot and hand, and the tumor size and depth of infiltration were not significantly unusual. Therefore, the recurrence and metastasis of CVC and their clinical and histological correlations remain to be further studied.

In summary, we presented a large group of patients with CVC to better characterize clinicopathological features and prognosis. The foot and lower legs are the most common sites where warty plaques or masses should be noted in clinical practice. The typical histopathological feature of CVC is an exo-endophytic growth pattern with blunt reticular ridges and pushing edges, but other clinical and pathologic characteristics are useful to distinguish CVC from its mimics.

## Data availability statement

The original contributions presented in the study are included in the article/supplementary material. Further inquiries can be directed to the corresponding author.

## Ethics statement 

Ethical approval was given by the Medical Ethics Committee, the First Affiliated Hospital of Chongqing Medical University (2022-150). The patients/participants provided their written informed consent to participate in this study.

## Author contributions

QY and SF were responsible for the conception and design of the study. QY, LH, and MJ were responsible for the literature screening, article selection, and data extraction. L-JD were responsible for the interpretation of data. QY and SF were responsible for statistical analysis. QY and SF were responsible for manuscript preparation. QY, LH, SF, L-JD, and MJ critically revised the manuscript. All authors read and approved the final manuscript.

## Acknowledgments

The patients in this study have given written informed consent to the publication of their case details.

## Conflict of interest

The authors declare that the research was conducted in the absence of any commercial or financial relationships that could be construed as a potential conflict of interest.

## Publisher’s note

All claims expressed in this article are solely those of the authors and do not necessarily represent those of their affiliated organizations, or those of the publisher, the editors and the reviewers. Any product that may be evaluated in this article, or claim that may be made by its manufacturer, is not guaranteed or endorsed by the publisher.
